# Relationships between PrP^Sc^ Stability and Incubation Time for United States Scrapie Isolates in a Natural Host System

**DOI:** 10.1371/journal.pone.0043060

**Published:** 2012-08-14

**Authors:** Catherine E. Vrentas, Justin J. Greenlee, Trudy L. Tatum, Eric M. Nicholson

**Affiliations:** National Animal Disease Center, Agricultural Research Service, United States Department of Agriculture, Ames, Iowa, United States of America; Creighton University, United States of America

## Abstract

Transmissible spongiform encephalopathies (TSEs), including scrapie in sheep (*Ovis aries*), are fatal neurodegenerative diseases caused by the misfolding of the cellular prion protein (PrP^C^) into a â-rich conformer (PrP^Sc^) that accumulates into higher-order structures in the brain and other tissues. Distinct strains of TSEs exist, characterized by different pathologic profiles upon passage into rodents and representing distinct conformations of PrP^Sc^. One biochemical method of distinguishing strains is the stability of PrP^Sc^ as determined by unfolding in guanidine hydrochloride (GdnHCl), which is tightly and positively correlated with the incubation time of disease upon passage into mice. Here, we utilize a rapid, protease-free version of the stability assay to characterize naturally occurring scrapie samples, including a fast-acting scrapie inoculum for which incubation time is highly dependent on the amino acid at codon 136 of the prion protein. We utilize the stability methodology to identify the presence of two distinct isolates in the inoculum, and compare isolate properties to those of a host-stabilized reference scrapie isolate (NADC 13-7) in order to assess the stability/incubation time correlation in a natural host system. We demonstrate the utility of the stability methodology in characterizing TSE isolates throughout serial passage in livestock, which is applicable to a range of natural host systems, including strains of bovine spongiform encephalopathy and chronic wasting disease.

## Introduction

Prion diseases, including bovine spongiform encephalopathy (BSE) in cattle, scrapie in sheep and goats, chronic wasting disease (CWD) in cervids, and Creutzfeldt-Jakob disease (CJD) in humans, are neurodegenerative diseases that manifest as behavioral changes and/or neurological signs and eventually progress to death. Also known as transmissible spongiform encephalopathies (TSEs), prion diseases are caused by misfolding of the cellular, protease-sensitive prion protein (PrP^C^) into an infectious, more protease-resistant form (PrP^Sc^) that accumulates in the nervous system and certain other body tissues.

Incubation time and susceptibility of animals to natural prion disease are affected by sequence variations in the prion gene (*PRNP*). In sheep, the presence of valine (V; as opposed to alanine, A) at codon 136 and the presence of glutamine (Q; as opposed to arginine, R) at codon 171 of the prion protein are associated with scrapie susceptibility [1], [2], [3], [4]. The capacity of (infectious) PrP^Sc^ to induce conformational changes in host PrP^C^ molecules varies depending on the host PrP amino acid sequence [5]. For example, the recombinant V_136_Q_171_ sheep prion protein has a higher intrinsic stability (which could lead to a lower PrP clearance rate in the cell), more compact structure, and a more â-rich folding intermediate than observed for recombinant PrP of more scrapie-resistant genotypes [6], [7]. Therefore, it is proposed that these properties are the molecular basis of differential scrapie susceptibility [7]. However, how the stability of the recombinant PrP (a model for cellular PrP^C^) correlates with the stability of the disease-associated PrP^Sc^ structures in the brain is not well-characterized.

Distinct strains of TSEs have been demonstrated to exist, manifesting as differences in the incubation period and in the profile of lesions and PrP^Sc^ accumulation in the brain upon experimental transmission to rodent hosts [8], [9], [10]. Based on the “protein only” hypothesis of prion infectivity [11], these strain properties must be the result of differences in the structure of PrP^Sc^, since the PrP amino acid sequence would be the same in each host [12], [13]. The structure of the PrP^Sc^ strain in the inoculum would be transmitted upon conversion of host PrP^C^ to PrP^Sc^ [14]. While over 20 different strains of sheep scrapie have been identified in the UK, the diversity of strains present in the US sheep population is not as well-characterized [15], and the level of diversity may be underestimated by the standard, non-transgenic mouse bioassay [16]. Since sheep hosts of different *PRNP* genotypes differ in their level of susceptibility to isolates of scrapie [1], and scrapie eradication efforts are based on breeding genetics, a better understanding of the properties of the different strains will enhance options for control and eradication of scrapie.

While rodent infection studies are the “gold standard” for strain characterization, prion strains can also be differentiated biochemically. Strains may exhibit differences in the profile of glycosylated, proteinase K (PK)-digested PrP^Sc^ isoforms as detected on a Western blot [17]. Alternatively, strains can be characterized by their stability as determined by denaturant unfolding using guanidine hydrochloride (GdnHCl) [12]. To measure stability, infected brain homogenate (BH) or enriched PrP^Sc^ is incubated with increasing concentrations of GdnHCl, and the level of PrP^Sc^ remaining is measured by methods such as PK digestion (unfolded PrP^Sc^ is degraded by the PK treatment along with the PrP^C^) coupled to Western blotting or ELISA; the conformation-dependent immunoassay [18]; or a method that quantitates changes in PrP^Sc^ solubility by centrifugation and Western blotting [19]. Here, we utilize a commercial ELISA (IDEXX HerdChek) with a non-antibody capture surface that binds PrP^Sc^ (but not PrP^C^ or unfolded PrP) to monitor the fraction of PrP^Sc^ remaining at each [GdnHCl]. This assay is denoted here as a “stability assay”; however, we are monitoring the irreversible denaturation of PrP^Sc^ [12], and the assay does not yield thermodynamic parameters for the unfolding process. Whether the assay is assessing loss of tertiary structure of PrP^Sc^, disruption of quaternary structure, or both is not known [20]. PrP^Sc^ stability is quantified as the GdnHCl concentration leading to half-maximal denaturation of the protein complexes ([GdnHCl]_1/2_).

The incubation time of prion strains intracranially (IC) inoculated into mice is directly correlated with the stability of PrP^Sc^ recovered from infected mouse brain, as measured by a version of the assay described above [20]. Lower stability has been linked to shorter sizes of mammalian PrP^Sc^ fibrils; thus, it is proposed that low stability leads to increased PrP^Sc^ fibril fragmentation, which decreases incubation times by facilitating the PrP^C^ PrP^Sc^ conversion [21], [22]. In contrast, hamster-adapted prion strains exhibit an opposite relationship, with short incubation times correlated with higher conformational stability; the short incubation time hamster strains are associated with decreased clearance from neurons [18], [23]. The stability assay has primarily been utilized to characterize rodent-passaged strains (of natural or synthetic prions; [20], [24]) or CJD PrP^Sc^ [25], [26], although recent studies have demonstrated a large difference in stability between atypical/Nor98 scrapie and classical scrapie in naturally infected sheep [19], [27].

Here, we utilized the ELISA-based stability assay to consider the relationships between PrP^Sc^ stability, incubation time, and host genotype in a natural host system for prion disease: sheep (*Ovis aries*) experimentally infected with classical scrapie. We characterized PrP^Sc^ from sheep infected with a fast-acting scrapie inoculum exhibiting host *PRNP* (codon 136) genotype dependence on incubation time, using the stability assay to provide molecular evidence for two separate isolates of scrapie and to compare their properties to those of a representative scrapie isolate (NADC 13-7). In addition, we use the stability approach to characterize the molecular properties of field scrapie PrP^Sc^ through the process of strain stabilization over the course of four serial passages in sheep.

## Results and Discussion

### Relationship between Genotype, Incubation Time, and PrP^Sc^ Stability in a V_136_-Dependent Scrapie Inoculum

As a model system for examining correlations between incubation time and PrP^Sc^ stability, we began by considering the properties of a United States sheep scrapie inoculum that exhibited a strong dependence of host genotype on the disease incubation time. The properties of the inoculum were originally noted in an experimental oral infection study of sheep with QQ genotypes at *PRNP* codon 171 (QQ_171_) and varying *PRNP* genotypes at codon 136 (AV_136_ or AA_136_). AV_136_ sheep developed disease with an incubation time of only 9–11 months [28], uncharacteristically short for oral inoculation of classical scrapie [29]. Similarly, when the same inoculum (denoted as “x124” and derived from an infected sheep herd in Idaho) was intracranially (IC) introduced into a separate population of QQ_171_ sheep, sheep with AV_136_ or VV_136_ genotypes progressed to clinical disease significantly faster than sheep with the AA_136_ genotype ([30]; Table 1). An AV_136_QR_171_ sheep was also susceptible to x124 IC infection with an incubation time of only 5.6 months [30], despite the fact that QR_171_ sheep are thought to be highly resistant to scrapie infection [2]. (Note that incubation times of scrapie in IC-infected sheep are shorter than incubation times in orally infected sheep; [29]).

**Table 1:** Clinical information for sheep samples.

Sample #	Breed	Genotype	Inoculum	Route of inoculation	Incubation time(months post-inoculation)	Ear tag #
1	Cheviot	AA_136_QQ_171_	x124	IC	16.5	**709**
2	Cheviot	AA_136_QQ_171_	x124	IC	16	**722**
3	Suffolk	AA_136_QQ_171_	x124	IL†	18	**3502**
4	Suffolk	AA_136_QQ_171_	x124	IC	26	**3504**
5	Suffolk	AA_136_QQ_171_	x124	IC	15	**3509**
6	Cheviot	AV_136_QQ_171_	x124	IC	6	**721**
7	Cheviot	AV_136_QQ_171_	x124	IC	5.5	**729**
8	Cheviot	VV_136_QQ_171_	x124	IC	5	**720**
9	Cheviot	VV_136_QQ_171_	x124	IC	4.5	**744**
10	Suffolk	AA_136_QQ_171_	136-VDEP	IC	16.5	**809**
11	Suffolk	AA_136_QQ_171_	136-VDEP	IC	16.5	**842**
12	Suffolk	VV_136_QQ_171_	13-7	IN‡	26	**813**
13	Suffolk	VV_136_QQ_171_	13-7	IN	27	**814**
14	Suffolk	AA_136_QQ_171_	13-7	IN	21	**822**
15	Suffolk	AA_136_QQ_171_	13-7	IN	17	**823**
16	Suffolk	VV_136_QQ_171_	136-VDEP	IN	7	**821**
17	Suffolk	VV_136_QQ_171_	136-VDEP	IN	7	**824**
18	Suffolk	AV_136_QQ_171_	136-VDEP	IN	10	**844**
19	Suffolk	AV_136_QQ_171_	136-VDEP	IN	14	**810**
20	Suffolk	AA_136_QQ_171_	13-7	IC	11	**831**
21	Suffolk	AA_136_QQ_171_	13-7	IC	10	**843**
22	Suffolk	AA_136_QQ_171_	None-control	N/A	End of study	**828**
23	Suffolk	AA_136_QQ_171_	None-control	N/A	End of study	**839**

† Sheep #3 was inoculated with x124 intralingually (IL) [34].

‡ IN = Intranasal/nasopharyngeal route of inoculation, resulting in contact with nasal mucosa and near immediate swallowing with no need for insertion of an esophageal tube.

**Figure 1:**
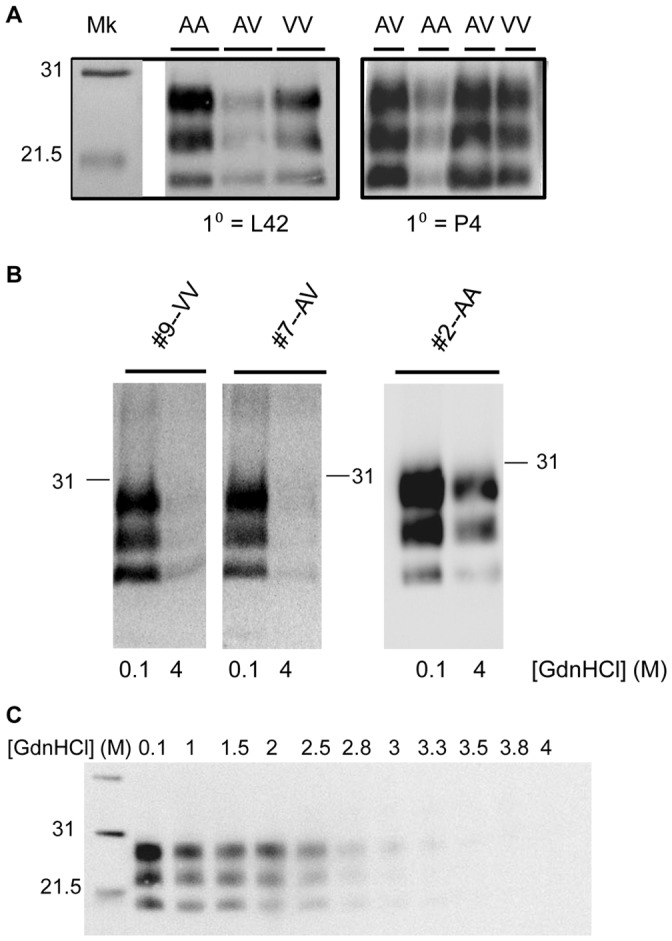
Western blotting patterns of PrP^Sc^ from x124-infected sheep brains. Each lane represents PK-digested PrP^Sc^ probed with an anti-PrP monoclonal antibody. Molecular weights (in kDa) of selected bands of a biotinylated protein molecular weight marker (Mk) are denoted in each panel. (A). *Comparison of banding patterns of PrP^Sc^ from x124-infected sheep of different genotypes*. Western blots were probed with either L42 (left) or P4 (right) primary antibody. Amounts of BH loaded per lane, from left, in mg equivalents of brainstem tissue: L42–0.2 mg, 0.2 mg, 0.4 mg; P4–0.4 mg, 0.1 mg, 0.4 mg, 0.4 mg. Genotype at codon 136 of sheep samples used is noted above the lanes. (B). and (C). *Western blots of GdnHCl-treated samples*. In (B), brainstem samples from the indicated sheep were treated at either 0.1 or 4 M GdnHCl as described in Methods; in (C), samples from a VV_136_ sheep (#9) were treated with concentrations ranging from 0.1 to 4 M GdnHCl, as indicated.

We examined the appearances of the x124-infected, PK-digested AA_136_, AV_136_, and VV_136_ PrP^Sc^ on a Western blot probed with P4 primary antibody (Fig. 1A, right panel); consistent with the previously published results, the Western blotting pattern of PK-digested PrP^Sc^ was not genotype-dependent [30]. We additionally observed that PrP^Sc^ from each genotype reacted with both P4 and L42, consistent with sheep classical scrapie strains as opposed to sheep BSE (Fig. 1A, left panel). AA_136_ and VV_136_ PrP^Sc^ exhibited similar tolerance to digestion with concentrations of PK ranging from 5 to 100 µg/ml (data not shown).

As an initial comparison of the stability of PrP^Sc^ in x124-infected sheep of each genotype, sheep brainstem samples were treated with either 0.1 or 4 M GdnHCl, and the amount of PrP^Sc^ remaining after PK digestion was examined by Western blot (Fig. 1B). PrP^Sc^ from AA_136_ sheep was relatively resistant to treatment with 4M GdnHCl; in contrast, PrP^Sc^ from AV_136_ and VV_136_ sheep was completely unfolded at 4 M GdnHCl. The reduction in PrP^Sc^ at more intermediate GdnHCl concentrations is depicted for a VV_136_ sheep in Fig. 1C.

**Figure 2:**
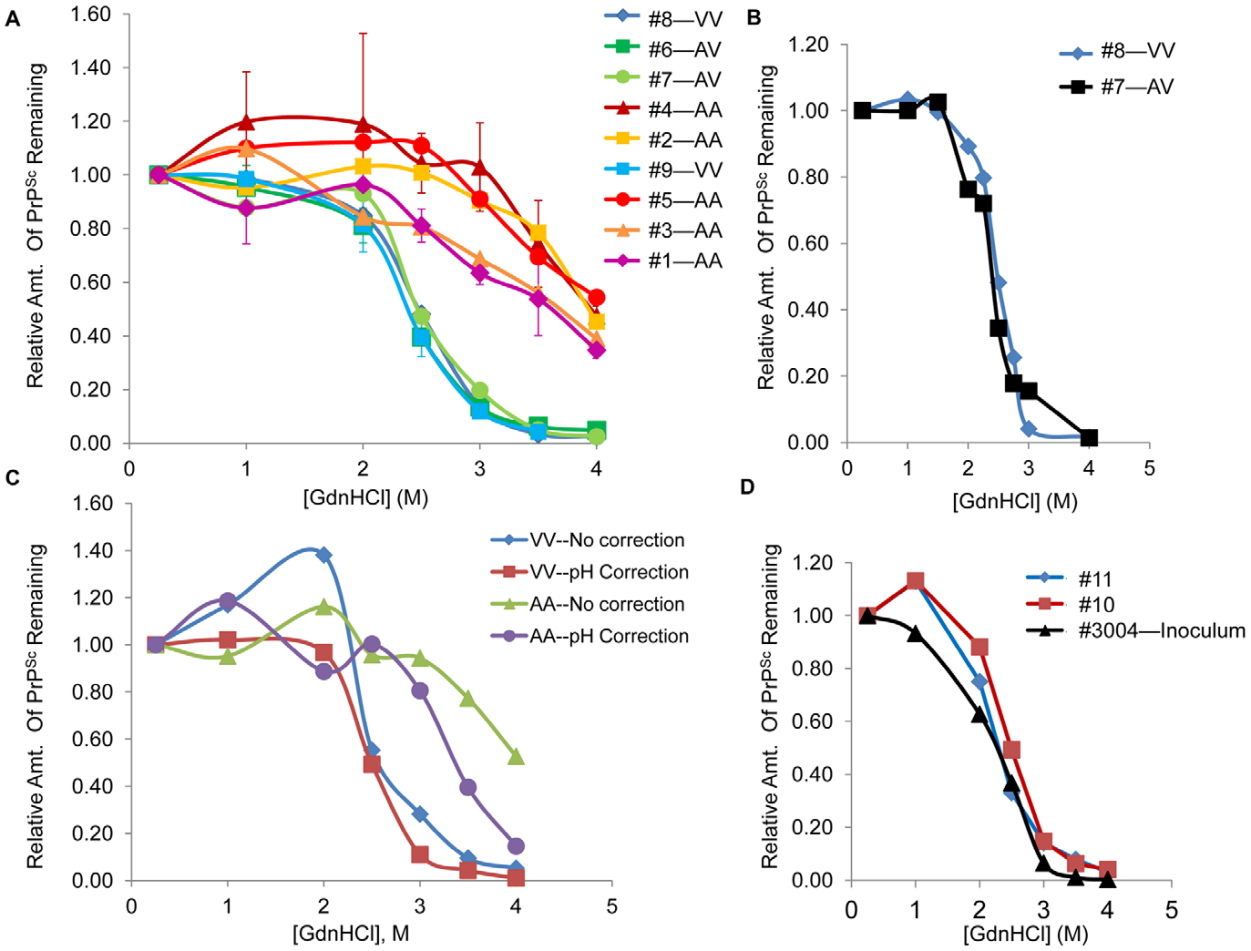
Unfolding Curves of 136-VDEP Passage Experiments. In each panel, GdnHCl unfolding curves were generated for PrP^Sc^ from the brainstem of the sheep samples indicated as described in Methods. In (A) and (D) only, curves were performed without adjustment of final GdnHCl solution pH, as described in Results. Points were normalized to the value at 0.25 M for each experiment. (A). *Comparison of x124-infected sheep of different genotypes*. Points represent averaged normalized values from 2–3 independent curves for each animal. Error bars display the standard deviation (SD) of 3 technical replicates for representative sheep samples of each genotype (sheep #1, #4, #6, #9). Technical replicates represent separate curves performed on separate days. Lines were used to connect data points and to estimate [GdnHCl]_1/2_; observed variations in sample baseline or deviations from a sigmoidal curve may represent real effects in these assays (i.e., no theoretical basis to expect a perfect sigmoidal curve). Average [GdnHCl]_1/2_ values were calculated for each animal and then averaged across genotypes; average [GdnHCl]_1/2_ values ± standard error of the mean (SEM) were as follows, as reported in the text: AA_136_ animals (n = 5 biological replicates)–3.8 M ±0.1 (approximate value due to partial curves); V_136_-containing animals (n = 4 biological replicates)–2.4 M ±0.02. More precise values for the [GdnHCl]_1/2_ of the AA_136_ animals were measured for three different animals (sheep #1, 3, and 4) under pH-adjusted conditions (Fig. 2C). Two x124-infected AV_136_ Suffolk sheep [34] also had PrP^Sc^ unfolding curves matching those for the AV_136_ Cheviot sheep depicted here (data not shown). (B). *Comparison of AV_136_ and VV_136_ PrP^Sc^ from the x124 infection experiment*. (C). *Characterization of the effect of pH on unfolding curves.* Blue curve–VV_136_ PrP^Sc^, no pH adjustment; Red–VV_136_, pH adjusted to neutral; Green–AA_136_, no pH adjustment; Purple–AA_136_, pH adjusted to neutral.(D). *Results of second passage of 136-VDEP into AA_136_ sheep*. For (B)-(D), the curves displayed represent data from a single experiment that was repeated at least two additional times in independent experiments (with comparable results in each repeat).

To better quantitate this effect, we used the HerdChek ELISA to directly measure the level of PrP^Sc^ remaining after treatment with increasing concentrations of GdnHCl (Fig. 2A). This version of the stability assay is more time-effective than other methods; after treatment with GdnHCl, only a simple dilution step is required before the ELISA, which can be completed in less than a day (a post-dilution [GdnHCl] of 0.25 M was selected as a compromise between the sample dilution factor and the ELISA signal; see Methods). Importantly, by avoiding a PK digestion step, this version of the ELISA-based stability assay provides a complete picture of the PrP^Sc^ present in the sheep brain, including PrP^Sc^ that is more protease-sensitive (if present; [31]). Similarly, we utilize the entire population of PrP^Sc^ in the PBS-homogenized brainstem tissue, as opposed to an enriched/purified fraction that may exclude certain populations of PrP^Sc^, such as lower-molecular weight aggregates that may not pellet during centrifugation. For AV_136_ and VV_136_ brain homogenates, the amount of PrP^Sc^ remaining plotted against the GdnHCl concentration approximated a sigmoidal curve (as described for mouse brain PrP^Sc^; [20]) with an average [GdnHCl]_1/2_ (± standard error of the mean) of 2.4 M ±0.02 for V_136_-containing sheep (n = 4 biological replicates). In contrast, the AA_136_ PrP^Sc^ was significantly more stable, with an estimated [GdnHCl]_1/2_ of 3.8 M ±0.1 overall (n = 5 biological replicates; value is estimated due to the high stability of the samples and the technical parameters of the assay). The unfolding curve of AV_136_ matched that of VV_136_ PrP^Sc^ when compared in more detail at the transition region of the curve (Fig. 2B). Based on the VV_136_ vs. AA_136_ difference observed in Fig. 2A, we hypothesize that only the V_136_ PrP in the heterozygous brains is converted to PrP^Sc^, and that the slightly longer incubation time of the AV_136_ versus VV_136_ sheep (Table 1) is due to the lower concentration of V_136_ PrP available in the brain for conversion. This is consistent with the findings of Jacobs et al. [32] for the conversion of only the V_136_Q_171_ PrP^C^ to PrP^Sc^ in AV_136_QR_171_ sheep. Therefore, on the first passage, relative stability was correlated with the incubation times that were in turn correlated with the genotypes.

We observed that the concentration of GdnHCl required in the more stable cases exceeded the buffering capacity of the PBS solution, resulting in a reduction in the final pH of samples as the [GdnHCl] was increased. Based on this observation, we compared unfolding curves for AA_136_ and VV_136_ PrP^Sc^ generated by the above method to curves generated with GdnHCl solution that had been adjusted to a neutral pH (Fig. 2C). We observed a pH-dependent change in the profile at 3–4 M GdnHCl (with an increased ELISA signal for acidic samples), but the difference in stability profile between AA_136_ and VV_136_ animals was observed with both methods. To examine the source of the pH effect on the ELISA signal, we treated 1 µl BH with 9 µl PBS adjusted to pH≈4 (treatment #1) or with PBS pH 7.4 (treatment #2) for 1 hour in the absence of GdnHCl. After dilution of samples with PBS (pH 7.4) up to 160 µl total volume, as in the above assays, we did not observe that treatment with acid increased the ELISA signal; instead, the signal was very slightly decreased in treatment 1 as compared to treatment 2. This result confirms that it is the pH in the presence of the GdnHCl that impacts the process of irreversible denaturation (here, reducing the level of denaturation at low pH). This observation is an important technical note for future studies of highly stable strains, as 1X PBS buffer is frequently used for preparation and testing of tissue homogenates.

### Observed Stability Differences are not Due to Inherent Effects of PrP^Sc^ Amino Acid Sequence

**Figure 3:**
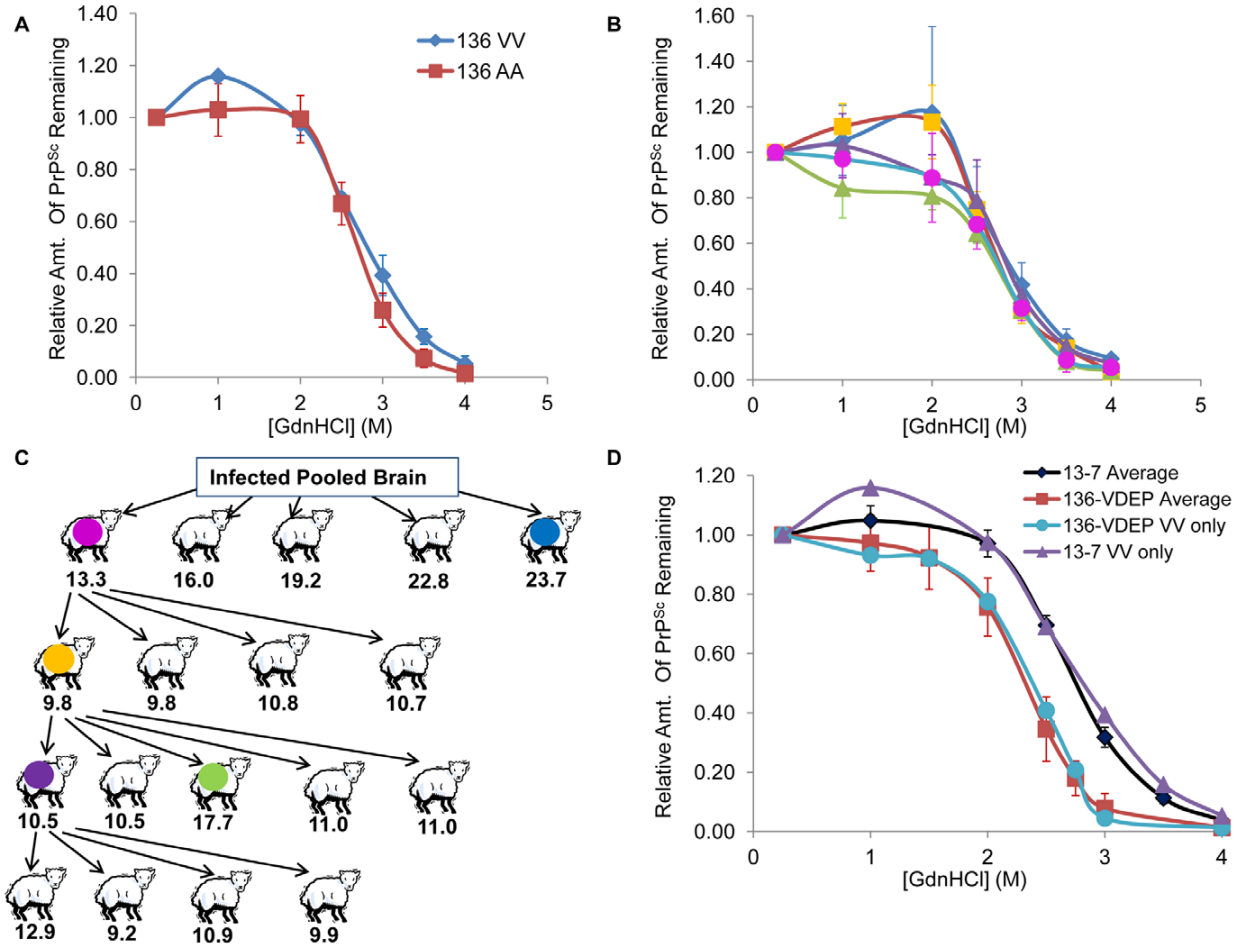
Comparison of 136-VDEP and 13-7 Scrapie Isolates. Curves were prepared as described in Fig. 2, with pH-adjusted GdnHCl. (A). *Comparison of 13-7 scrapie stability in AA_136_ and VV_136_ hosts*. Red curve–13-7- infected PrP^Sc^ from AA_136_ hosts (average of sheep #14, #15, #20); Blue curve–13-7-infected PrP^Sc^ from VV_136_ hosts (average of sheep #13, 14). The results from three technical replicates from each animal were averaged before averaging biological replicates; red error bars represent SEM and blue error bars represent the range. (B, C). *Serial passage of 13-7 inoculum in Suffolk sheep.* (C) depicts the process of serial passage, with incubation times of disease in the hosts indicated below the animal in months. Colored symbols in (C) correspond to the colors used to depict the unfolding curve of PrP^Sc^ from the same sheep in (B). In (B), the results from four independent curves from each animal were averaged, and error bars reflect the SD for technical replicates. (D). *Comparison of stability between 136-VDEP and 13-7 isolates*. Red curve–136-VDEP-infected PrP^Sc^; Black curve–13-7-infected PrP^Sc^. Curves are averaged across 4 (red) or 8 (black) biological replicates of each isolate, and error bars reflect SEM of biological replicates. To allow for statistical analysis, the 136-VDEP curve includes AV_136_ and VV_136_ animals, and the 13-7 curve includes VV_136_ and AA_136_ animals (since Fig. 3A demonstrates that genotype does not have an inherent effect on stability). The same pattern is observed with averaged curves of two 136-VDEP-infected VV_136_ sheep (blue curve) and two 13-7-infected VV_136_ sheep (purple curve). [GdnHCl]_1/2_ values for the individual animals were also calculated and averaged, with a mean [GdnHCl]_1/2_ for 136-VDEP of 2.36 M±0.06 (SEM) and a mean [GdnHCl]_1/2_ for 13-7 of 2.80 M±0.10 (SEM). Means of the two populations were significantly different by an unpaired student’s T-test (*P*-value = 0.0003).

The observed difference in stability between the x124-infected AA_136_ and VV_136_ sheep PrP^Sc^ could be explained by two hypotheses: either (1) the stability of the host scrapie PrP^Sc^ formed from a singleisolate in the x124 inoculum is host genotype-dependent, or (2) multiple, distinct isolates of scrapie were present in the flock that served as the source of the scrapie inoculum (and the AA_136_ sheep brains predominantly accumulated a different isolate than did the other sheep). In order to examine if there is an effect of host protein amino acid sequence on the stability of PrP^Sc^, we utilized samples of sheep brainstem experimentally infected with the same classical scrapie isolate, NADC 13-7 (more details in Methods; Table 1). The ability to compare samples that have been infected with a single isolate of host-stabilized scrapie provides a superior system to analysis of field samples of different genotypes, which may be infected with different, or mixed, strains/isolates. The stability of PrP^Sc^ from 13-7 infected AA_136_ sheep (#14, #15, #20) and the stability of PrP^Sc^ from 13-7 infected VV_136_ sheep (#12, #13) were compared. Despite apparent differences in the incubation time of the two AV_136_ and the two VV_136_ animals (Table 1), we did not observe differences in the stability of PrP^Sc^ from the two different host genotypes at the level of resolution of this assay (Fig. 3A). This result also demonstrates the potential use of this methodology for comparisons of the effects of *PRNP* polymorphisms on PrP^Sc^ properties in natural hosts.

### Identification of Distinct Scrapie Strains in the Genotype-dependent x124 Scrapie Inoculum

The results of the 13-7-infected host comparison suggest that the data in Fig. 2A could alternatively be explained by the presence of more than one isolate in the x124 inoculum. Analysis of the results of a second round of sheep inoculations allowed us to confirm this hypothesis. BH from a single AV_136_QR_171_ sheep (#3004) infected with x124 was IC inoculated into two AA_136_QQ_171_ sheep (#10 and #11), each of which developed clinical signs of scrapie and accumulated PrP^Sc^ in the brainstem (Table 1). The unfolding curves of PrP^Sc^ from sheep #10 and #11 were consistent with that of the #3004 inoculum (Fig. 2D), with [GdnHCl]_1/2_ values consistent with those of the VV_136_ (and not the AA_136_) sheep from the first passage of the pooled x124 inoculum (Fig. 2A, C). This result suggests that at least two different isolates of scrapie were present in the original x124 inoculum. One, characterized by a [GdnHCl]_1/2_ of 2.3–2.4 M in this assay, is fast-acting in sheep carrying a V_136_ allele, but is also capable of causing disease in AA_136_ sheep by IC inoculation. We refer to this isolate as “136-VDEP.” The other isolate is predominant in the x124-infected AA_136_ sheep brain and exhibits higher stability in GdnHCl (approximately 3.3–4 M, depending on the discussed pH effect). It is possible that the x124-infected AA_136_ sheep have accumulated both forms of PrP^Sc^ in their brainstem (we speculate that differences in the curves between individual AA_136_ sheep could be due to differences in the level of 136-VDEP that accumulated in the brain). The stability profile of AA_136_ PrP^Sc^ from x124-infected sheep is consistent with reports that classical field scrapie samples are stable up to 3–4 M GdnHCl as measured by PK-Western dot blot [27].

Note that while the stability of their PrP^Sc^ was low, the incubation times for sheep #10 and #11 (AA_136_) were relatively long; VV_136_ sheep infected with the same dose of brain #3004 intranasally (a slower-onset method of inoculation) succumbed to scrapie in less than half the time in the same experiment (Table 1). We attribute this to a host PrP^C^ genotype influence on disease kinetics, an effect that must supersede the role of PrP^Sc^ complex stability in determining incubation time in this case. As compared to inoculation studies of isogenic mouse hosts, explorations of incubation time relationships in livestock must take into consideration the role of both host (PrP^C^) genotype and PrP^Sc^ stability.

### Molecular Typing of Serially Passaged US Scrapie Inoculum 13-7

The results of this study demonstrate the utility of the stability assay in distinguishing between prion isolates which have apparently identical Western blot profiles. Often, prion isolates are serially passaged in order to select and stabilize an isolate. As an additional application of the ELISA-based stability assay to livestock systems, we used stability profiles to investigate the composition of PrP^Sc^ from sheep brains during serial passage of the 13-7 strain. In previous work [33], the initial 13-7 pooled brain inoculum (pool of 13 sheep brains from 7 different flocks) was used to IC infect 5 different Suffolk lambs (all AA_136_); incubation times before development of clinical symptoms varied between 13 and nearly 24 months (Fig. 3C). For each passage, BH (1 ml of a 10% w/v brain suspension) from the sheep with the shortest incubation time was used to inoculate the lambs in the subsequent passage [33]; average incubation times for each passage were Passage 1 (P1) = 19.0 mo., P2 = 10.3 mo, P3 = 12.1 mo., and P4 = 10.7 mo. We determined the stability of PrP^Sc^ from selected lambs from each passage to determine if the relative [GdnHCl]_1/2_ tracked with the incubation time of the sheep (Fig. 3B). Significant differences in the incubation time of sheep at the first passage and the incubation time at later passages were present, suggesting stabilization of the isolate subsequent to the first passage (*P*-values were 0.0007, 0.003, and 0.001 when comparing passage 1 incubation time with incubation time of each of the subsequent passages (P2–P4), respectively). However, we did not observe a relationship between the incubation time of the sheep and the [GdnHCl]_1/2_. While we observe some variations in the upper baseline, the rest of the curve is very similar for the different samples (Fig. 3B). This result is consistent with the fact that the lesion profiles and PrP^Sc^ immunohistochemistry (IHC) patterns in brain sections did not differ between subpassage groups [33]. It is possible that multiple isolates were present in the original inoculum, but the data suggests that on the first passage, the predominant isolate in each of the infected sheep was the same. This in turn suggests that other factors besides the specific isolate of scrapie that is present influenced the incubation time and caused the decrease in incubation time between the first and the subsequent passages. One possibility is that dosage of the predominant scrapie isolate increased upon passage. While we did not observe differences in PrP^Sc^stability in this case study, we note that this would be a useful and rapid method for directly tracking prion isolates in other livestock prion stabilization studies.

### Correlation between PrP^Sc^ Stability and Incubation Time in a Natural Host System

As discussed, in two independent experimental inoculations of sheep with homogenate containing 136-VDEP [28], [30], rapid disease progression was observed in sheep carrying at least one V_136_ allele. Similar rapid disease development (incubation times of ≈7 months for VV_136_ animals) was observed upon intranasal inoculation of AV_136_ and VV_136_ sheep with a brain from a single 136-VDEP-infected AV_136_QR_171_ animal (Table 1). In the same study, sheep intranasally infected with (stabilized) 13-7, serving as a reference isolate of U.S. sheep scrapie, progressed to disease in an average of 22 months, in a codon 136 genotype-independent fashion (Table 1). We suggest that the dose of 13-7 in this study was overwhelming since 100% (4/4) of the inoculated sheep developed clinical signs and accumulation of brainstem PrP^Sc^ due to inoculation with this passaged and host-adapted strain (Table 1, sample #’s 12–15). Therefore, experimental evidence combined over multiple studies in sheep suggests that the incubation time of the stabilized 13-7 isolate is longer than that of the 136-VDEP isolate (which has an unusually short incubation time for classical scrapie in sheep).

We utilized PrP^Sc^ stability information from the experiments described above to determine if the 13-7 and 136-VDEP isolates also exhibited a significant difference in stability. Unfolding curves from Fig. 3A and Fig. 3B (excluding the first passage in Fig. 3B; n = 8) were averaged to generate a 13-7 unfolding curve, and (pH-controlled) unfolding curves from AV_136_ and VV_136_ sheep (n = 4) were averaged to generate a 136-VDEP unfolding curve (Fig. 3D). The shorter incubation time of 136-VDEP in sheep correlates with a lower stability (lower [GdnHCl]_1/2_ value), suggesting that the correlation between incubation time and PrP^Sc^ stability observed in mouse-passaged samples [20] may also apply to natural hosts. We note that the incubation time and stability also appear to be correlated for the isolate present in the x124-infected AA_136_ sheep (average of 19 months for the IC samples investigated here, [GdnHCl]_1/2_>3; compared to an average of 5 months for the IC-inoculated VV_136_ sheep for 136-VDEP and an average of 11 months for IC-inoculated AA_136_ sheep for 13-7; [33] and Table 1). However, it is possible that the x124 inoculum, which also carried the 136-VDEP isolate, only carried a low dose of the higher-stability isolate. Further experimentation, in which x124-infected AA_136_ brain homogenate is passaged into additional sheep, is warranted to confirm the incubation time upon use of a higher dose. Interestingly, Bulgin et al. [28] report that the 136-VDEP isolate in orally-infected AV_136_ sheep led to an abnormally fine, “sprinkled” accumulation of PrP^Sc^ throughout the affected tissues. Therefore, it is possible that the relationship between diffuse aggregation, low PrP^Sc^ stability, and short incubation times observed in mice [34] also applies to strains of classical scrapie in the natural host. However, we note that in contrast, Hamir et al. (2009) did not observe differences in IHC PrP^Sc^ labeling of x124-infected brain sections of different genotypes.

In summary, we demonstrate the utility of the ELISA-based GdnHCl stability assay in the analysis of prion disease isolates (otherwise indistinguishable by the standard Western blotting test) in natural hosts. The commercial, non-PK-dependent ELISA utilized here is particularly simple and rapid for use in these measurements. We identify the presence of a mixture of isolates in an inoculum previously believed to reflect a single isolate of scrapie [30] and characterize field scrapie samples across the process of serial passage in sheep. We demonstrate that the short-incubation “136-VDEP” isolate is associated with an unstable PrP^Sc^ higher structure as compared to the longer-incubation, higher stability scrapie isolate13-7, analogous to the previously reported correlation in mouse-adapted strains [20]. The second passage of the 136-VDEP isolate into AA_136_ animals highlights the role of host genotype in this incubation time/stability correlation. Finally, we do not identify differences in the inherent stability of A_136_ PrP^Sc^ and V_136_ PrP^Sc^ (when infected by the same host-stabilizedprion isolate) in this assay of irreversible denaturation. The approach presented here is broadly applicable to naturally occurring TSEs in natural host species (with different PrP primary structure), and this work will inform future experiments designed to further our understanding of livestock TSE incubation time, genotype, and stability relationships.

## Materials and Methods

### Ethics Statement

All animal studies were carried out in accordance with the Guide for the Care and Use of Laboratory Animals (Institute of Laboratory Animal Resources, National Academy of Sciences, Washington, DC) and the Guide for the Care and Use of Agricultural Animals in Research and Teaching (Federation of Animal Science Societies, Champaign, IL); protocols were approved by the Institutional Animal Care and Use Committee at the National Animal Disease Center. Previously published tissue samples are cited under the heading Sheep Brain Sample Sources. Samples from ongoing studes are covered under protocol #3893.

### Sheep Brain Sample Sources

Scrapie-infected sheep brain samples were obtained from previous studies [28], [30], [33], [35] or ongoing pathogenesis studies at the National Animal Disease Center (protocol #3893). Briefly, x124-infected samples [30] (Table 1; all animals were RR_154_QQ_171_) were IC inoculated as lambs with the x124 inoculum, prepared from a pool of 7 scrapie-affected brains from animals that were QQ_171_ and either AA (n = 5), AV (n = 1), or VV (n = 1) at codon 136. Animals were allowed to progress to the incidence of clinical disease, euthanized, and necropsied upon incidence of clinical signs; the presence of PrP^Sc^ in the brain was confirmed by Western blotting and IHC [30], [35]. Sheep #3004 (AV_136_QR_171_) was orally inoculated with x124 (5ml of a 5% brain homogenate) and similarly treated [28].

For the comparison of 136-VDEP and (stabilized) 13-7 infection of sheep, lambs were intranasally (IN) inoculated with 1 ml of either 13-7 (derived from a single brain carrying the 13-7 isolate after stabilization via 4 passages in sheep) or 136-VDEP (derived from sheep brain #3004, as described) BH. Animals were euthanized upon development of clinical symptoms or at the end of the experiment (30 months). Two uninoculated control sheep did not develop clinical symptoms during the time frame of the experiment (sheep #22 and #23, Table 1).

Finally, samples for the molecular investigation of the serial passage of 13-7 in sheep were obtained from Hamir et al. [33]. Briefly, pooled BH denoted as “13-7” was prepared from 13 scrapie-infected sheep brains (derived from 7 different U.S. source flocks) and passaged in 4 generations of Suffolk lambs (predominantly AA_136_RR_154_QQ_171_). The incubation time data for serial passage of scrapie isolate 13-7 was analyzed by analysis of variance using a general linear model for unbalanced data (SAS for Windows, Version 9.2, SAS Institute Inc.,Cary, NC, USA). A significance level of 5% was also used for comparisons between passages.

### Preparation and Western Blotting of Sheep Brain Homogenates

Samples of the brainstem of sheep brains that had been frozen at −80°C or −20°C were used to prepare BH for Western blotting and ELISA analysis. Tissue samples were bead homogenized at 20% (w/v) in 1× PBS (Dulbecco’s PBS, pH 7.4, lacking calcium and magnesium) and tested shortly after storage at −80°C. PK digestion of whole BH was performed in 1X PBS for 1 hr. at 37°C, at a final [PK] of 100 µg/ml, followed by inactivation of PK with 1 mM Pefabloc (Roche). Samples were boiled in 1x LDS loading dye (Invitrogen) and 5% â-mercaptoethanol and loaded to 12% NuPAGE Bis-Tris gels (MOPS running buffer, Invitrogen); samples were transferred to PVDF and membranes were blocked for 30 minutes in TBST (Tris-Buffered Saline +0.1% Tween) +3% BSA. Membranes were successively probed with either the L42 or the P4 primary antibody (monoclonal mouse; 1∶10,000 for P4, 1∶500 for L42; overnight at 4°C), biotinylated sheep anti-mouse secondary antibody (1∶10,000, 45 minutes, room temperature), and HRP-conjugated streptavidin (1∶10,000, 45 minutes, room temperature), all in TBST +3% BSA. Blots were imaged with ECL Plus reagent (Pierce) by either chemiluminescence or fluorescence on a GBOX instrument (Synoptics).

To perform the Western blots of GdnHCl-treated samples (Fig. 1B, C), BH was incubated with 0.1 or 4 M GdnHCl (Fig. 1B) or concentrations of GdnHCl increasing to 4 M (Fig. 1C) for 1 hour, followed by dilution of samples with 1X PBS to a final [GdnHCl] of 0.1 M. Diluted samples were PK-digested and treated with Pefabloc as described above, followed by overnight precipitation at −20°C by the addition of 3–4 volumes of acetone. After recovery of the precipitate by centrifugation, the pellet was boiled in loading buffer and subjected to Western blotting with P4 antibody as described above.

### ELISA-based PrP^Sc^ Stability Assay

The IDEXX HerdChek BSE-Scrapie Antigen ELISA test kit was used to selectively detect the presence of PrP^Sc^ (as opposed to PrP^C^ or unfolded PrP^Sc^) in the sheep brains. BH from sheep brainstem samples, prepared as described above, were mixed with 1x PBS and between 0.25 M and 4 M final [GdnHCl] (the GdnHCl solution used here was prepared in 1x PBS). BH was diluted up to 8X into PBS in some cases in order to bring the final OD_450_ in the ELISA well to 1.0 or below. No genotype-dependent trends in the necessary sample dilution factor were present (data not shown), suggesting that the concentration of BH in the assay was not a significant variable in the differences noted between genotypes. The 20% BH is diluted at least 1 to 10 in the final sample volume of 10 µl. Samples were incubated in GdnHCl for approximately one hr. at room temperature (RT) and then diluted with PBS and GdnHCl to bring the final [GdnHCl] to 0.25 M in each sample. After dilution, samples were immediately loaded to ELISA plates from the kit, following the “short protocol” for small ruminants in the manufacturer instructions. Optical density (OD) values of each sample were read at 450 nm on a SpectraMax 190 plate reader. Values were normalized to the OD_450_ of the 0.25 M sample after subtraction of a negative control well (negative control provided by the IDEXX kit) as background.

## References

[1] Goldmann W, Hunter N, Smith G, Foster J, Hope J (1994) PrP genotype and agent effects in scrapie: change in allelic interaction with different isolates of agent in sheep, a natural host of scrapie. J Gen Virol 75: 989–995. 7909834 10.1099/0022-1317-75-5-989

[2] O’Rourke KI, Holyoak GR, Clark WW, Mickelson JR, Wang S, et al (1997) PrP genotypes and experimental scrapie in orally inoculated Suffolk sheep in the United States. J Gen Virol 78: 975–978. 9129673 10.1099/0022-1317-78-4-975

[3] Trianulis MA (2002) Influence of the prion protein gene, Prnp, on scrapie susceptibility in sheep. APMIS. 110: 33–43. 10.1034/j.1600-0463.2002.100105.x 12064254

[4] Detwiler LA, Baylis M (2003) The epidemiology of scrapie. Rev Sci Tech. 22: 121–143. 10.20506/rst.22.1.1386 12793776

[5] Bossers A, Belt PBGM, Raymond GJ, Caughey B, de Vries R, et al (1997) Scrapie susceptibility-linked polymorphisms modulate the in vitro conversion of sheep prion protein to protease-resistant forms. Proc Natl Acad Sci USA 94: 4931–4936. 9144167 10.1073/pnas.94.10.4931 PMC24608

[6] Rezaei H, Marc D, Choiset Y, Takahashi M, Hui Bon Hoa G, et al (2000) High yield purification and physic-chemical properties of full-length recombinant allelic variants of sheep prion protein linked to scrapie susceptibility. Eur J Biochem 267: 2833–2839. 10806380 10.1046/j.1432-1033.2000.01347.x

[7] Rezaei H., Choiset Y., Eghiaian F., Treguer E., Mentre P., Debey P., Grosclaude J., & Haertle T (2002) Amyloidogenic unfolding intermediates differentiate sheep prion protein variants. J Mol Biol 322, 799–814. 10.1016/s0022-2836(02)00856-2 12270715

[8] Fraser H, Dickinson AG (1968) The sequential development of the brain lesion of scrapie in three strains of mice. J Comp Pathol 78: 301–311. 4970192 10.1016/0021-9975(68)90006-6

[9] Bruce ME, McBride PA, Farquhar CF (1989) Precise targeting of the pathology of the sialoglycoprotein, PrP, and vacuolar degeneration in mouse scrapie. Neurosci Lett 102: 1–6. 2550852 10.1016/0304-3940(89)90298-x

[10] Hecker R, Taraboulos A, Scott M, Pan KM, Yang SL, Torchia M, Jendroska K, DeArmond SJ (1992) Prusiner SB (1992) Replication of distinct scrapie prion isolates is region specific in brains of transgenic mice and hamsters. Genes Dev 6: 1213–1228. 1628828 10.1101/gad.6.7.1213

[11] Prusiner SB (1982) Novel proteinaceous infectious particles cause scrapie. Science 216, 136–144. 10.1126/science.6801762 6801762

[12] Peretz D, Scott MR, Groth D, Williamson RA, Burton DR, et al (2001) Strain-specified relative conformational stability of the scrapie prion protein. Protein Sci 10: 854–863. 11274476 10.1110/ps.39201 PMC2373967

[13] Peretz D, Williamson RA, Legname G, Matsunaga Y, Vergara J, Burton, et al (2002) A change in the conformation of prions accompanies the emergence of a new prion strain. Neuron 34: 921–932. 12086640 10.1016/s0896-6273(02)00726-2

[14] Telling GC, Parchi P, DeArmond SJ, Cortelli P, Montagna P, et al (1996) Evidence for the conformation of the pathologic isoform of the prion protein enciphering and propagating prion diversity. Science 274: 2079–2082. 8953038 10.1126/science.274.5295.2079

[15] Bulgin MS, Melson SS (2007) What veterinary practitioners should know about scrapie. J Am Vet Med Assn 230: 1158–1164. 10.2460/javma.230.8.1158 17501652

[16] Thackray AM, Hopkins L, Lockey R, Spiropoulos J, Bujdoso R (2012) Propagation of ovine prions from “poor” transmitter scrapie isolates in ovine PrP transgenic mice. Exp Mol Pathol 92: 167–174. 22120785 10.1016/j.yexmp.2011.11.004

[17] Bessen RA, Marsh RF (1994) Distinct PrP properties suggest the molecular basis of strain variation in transmissible mink encephalopathy. J Virol 68: 7859–7868. 7966576 10.1128/jvi.68.12.7859-7868.1994 PMC237248

[18] Safar J, Wille H, Itri V, Groth D, Serban H, et al (1998) Eight prion strains have PrP^Sc^ molecules with different conformations. Nat Med 4: 1157–1165. 9771749 10.1038/2654

[19] Pirisinu L, Di Bari M, Marcon S, Vaccari G, D’Agostino C, et al (2010) A new method for the characterization of strain-specific conformational stability of protease-sensitive and protease-resistant PrP^Sc^ . PLoS One 5: e12723. 20856860 10.1371/journal.pone.0012723 PMC2939050

[20] Legname G, Nguyen H-O B, Peretz D, Cohen FE, DeArmond SJ, et al (2006) Continuum of prion protein structures enciphers a multitude of prion isolate-specified phenotypes. Proc Natl Acad Sci USA 103: 19105–19110. 17142317 10.1073/pnas.0608970103 PMC1748184

[21] Sun Y, Makarava N, Lee C-I, Laksanalamai P, Robb FT, et al (2008) Conformational stability of PrP amyloid fibrils controls their smallest possible fragment size. J Mol Biol 376: 1155–1167. 18206163 10.1016/j.jmb.2007.12.053 PMC2276463

[22] Zampieri M, Legname G, Altafini C (2009) Investigating the conformational stability of prion strains through a kinetic replication model. PLoS Computational Biol 5: e1000420. 10.1371/journal.pcbi.1000420 PMC2697384 19578427

[23] Ayers JI, Schutt CR, Shikiya RA, Aguzzi A, Kincaid AE, et al (2011) The strain-encoded relationship between PrP^Sc^ replication, stability and processing in neurons is predictive of the incubation period of disease. PLoS Pathog 7: e1001317. 21437239 10.1371/journal.ppat.1001317 PMC3060105

[24] Thackray AM, Hopkins L, Spiropoulos J, Bujdoso R (2008) Molecular and transmission characteristics of primary-passaged ovine scrapie isolates in conventional and ovine PrP transgenic mice. J Virol 82: 11197–11207. 18768980 10.1128/JVI.01454-08 PMC2573291

[25] Choi YP, Peden AH, Groner A, Ironside JW, Head MW (2010) Distinct stability states of disease-associated human prion protein identified by conformation-dependent immunoassay. J Virol 84: 12030–12038. 20844046 10.1128/JVI.01057-10 PMC2977900

[26] Kim C, Haldiman T, Cohen Y, Chen W, Blevins J, et al (2011) Protease-sensitive conformers in broad spectrum of distinct PrP^Sc^ structures in sporadic Creutzfeldt-Jakob disease are indicator of progression rate. PLoS Pathog 7: e1002242. 21931554 10.1371/journal.ppat.1002242 PMC3169556

[27] Wemheuer WM, Benestad SL, Wrede A, Schultze-Sturm U, Wemheuer WE, et al (2009) Similarities between forms of sheep scrapie and Creutzfeldt-Jakob disease are encoded by distinct prion types. Am J Pathol 175: 2566–2573. 19850886 10.2353/ajpath.2009.090623 PMC2789619

[28] Bulgin MS, Sorensen SJ, Matlock ME (2006) Association between incubation time and genotype in sheep experimentally inoculated with scrapie-positive brain homogenate. Am J Vet Res 67: 498–504. 16506916 10.2460/ajvr.67.3.498

[29] Hamir AN, Kunkle RA, Richt JA, Miller JM, Cutlip RC, et al (2005) Experimental transmission of sheep scrapie by intracerebral and oral routes to genetically susceptible Suffolk sheep in the United States. J Vet Diagn Invest 17: 3–9. 15690944 10.1177/104063870501700103

[30] Hamir AN, Richt JA, Kunkle RA, Greenlee J J, Bulgin MS, et al (2009) Characterization of a US sheep scrapie isolate with short incubation time. Vet Path 46: 1205–1212. 19605918 10.1354/vp.08-VP-0258-H-FL

[31] Sajnani G, Silva CJ, Ramos A, Pastrana MA, Onisko BC, et al (2007) PK-sensitive PrP is infectious and shares basic structural features with PK-resistant PrP. PLoS Pathogens 8: e1002547. 10.1371/journal.ppat.1002547 PMC3291653 22396643

[32] Jacobs JG, Bossers A, Rezaei H, van Keulen JM, McCutcheon S, et al (2011) Proteinase K-resistant material in ARR/VRQ sheep brain affected with classical scrapie is composed mainly of VRQ prion protein. J Virol 85: 12537–12546. 21917981 10.1128/JVI.00448-11 PMC3209378

[33] Hamir AN, Kunkle RA, Richt JA, Greenlee J J, Miller JM (2009) Serial passage of sheep scrapie inoculum in Suffolk sheep. Vet Pathol 46: 39–44. 19112113 10.1354/vp.46-1-39

[34] Bett C, Joshi-Barr S, Lucero M, Trejo M, Liberski P, et al (2012) Biochemical properties of highly neuroinvasive prion strains. PLoS Pathog 8: e1002522. 22319450 10.1371/journal.ppat.1002522 PMC3271082

[35] Hamir AN, Kunkle RA, Bulgin MS, Rohwer RG, Gregori L, et al (2008) Experimental transmission of scrapie agent to susceptible sheep by intralingual or intracerebral inoculation. Can J Vet Res 72: 63–67. 18214164 PMC2117369

